# Heparin treatment is associated with a delayed diagnosis of Alzheimer’s dementia in electronic health records from two large United States health systems

**DOI:** 10.1038/s41380-024-02757-5

**Published:** 2024-10-08

**Authors:** Benjamin Readhead, Eyal Klang, Undina Gisladottir, Maxence Vandromme, Li Li, Yakeel T. Quiroz, Joseph F. Arboleda-Velasquez, Joel T. Dudley, Nicholas P. Tatonetti, Benjamin S. Glicksberg, Eric M. Reiman

**Affiliations:** 1 ASU-Banner Neurodegenerative Disease Research Center, Arizona State University, Tempe, AZ 85281 USA; 2 Icahn School of Medicine at Mount Sinai, New York, NY 10029 USA; 3 Faculty of Medicine, Tel Aviv University, Tel-Aviv, Israel; 4 Department of Biomedical Informatics, Columbia University, New York, NY USA; 5 Sema4, Stamford, CT USA; 6 Departments of Psychiatry and Neurology, Massachusetts General Hospital and Harvard Medical School, Boston, MA USA; 7 Schepens Eye Research Institute of Mass Eye and Ear and Department of Ophthalmology, Harvard Medical School, Boston, MA USA; 8 Department of Computational Biomedicine, Cedars-Sinai Medical Center, Los Angeles, CA USA; 9 Samuel Oschin Cancer Center, Cedars-Sinai Medical Center, Los Angeles, CA USA; 10 Banner Alzheimer’s Institute, Phoenix, AZ 85006 USA

**Keywords:** Neuroscience, Diseases, Drug discovery

## Abstract

Recent studies suggest that heparan sulfate proteoglycans (HSPG) contribute to the predisposition to, protection from, and potential treatment and prevention of Alzheimer’s disease (AD). Here, we used electronic health records (EHR) from two different health systems to examine whether heparin therapy was associated with a delayed diagnosis of AD dementia. Longitudinal EHR data from 15,183 patients from the Mount Sinai Health System (MSHS) and 6207 patients from Columbia University Medical Center (CUMC) were used in separate survival analyses to compare those who did or did not receive heparin therapy, had a least 5 years of observation, were at least 65 years old by their last visit, and had subsequent diagnostic code or drug treatment evidence of possible AD dementia. Analyses controlled for age, sex, comorbidities, follow-up duration and number of inpatient visits. Heparin therapy was associated with significant delays in age of clinical diagnosis of AD dementia, including +1.0 years in the MSMS cohort (*P* < 0.001) and +1.0 years in the CUMC cohort (*P* < 0.001). While additional studies are needed, this study supports the potential roles of heparin-like drugs and HSPGs in the protection from and prevention of AD dementia.

## Introduction

Recent evidence suggests that the interaction between APOE and heparan sulfate proteoglycans (HSPGs) may be relevant to the predisposition to, protection from, and potential treatment and prevention of Alzheimer’s disease (AD). HSPG is located on the surface of all eukaryote cells and is known to bind to many different proteins. We recently found that apolipoprotein E isoforms bind with differential affinity to HSPG, corresponding to their suggested risk for AD (ApoE4 > 3 > 2), that it binds with extremely low affinity to the rare ApoE Christchurch variant which we found to be associated with an exceptionally low risk of AD dementia in a Presenilin 1 (PSEN1) E280A mutation carrier from the world’s largest autosomal dominant AD (ADAD) kindred, and that it binds with extremely low affinity to wild type APOE in the presence of an antibody bound to the ApoE Christchurch binding domain. HSPG Glypican-4 has been reported as a driver of ApoE4 induced tau hyperphosphorylation [1]. In other studies [2–5], HSPG has been suggested to help mediate neuronal uptake and trans-synaptic transmission of tau, the main component of neurofibrillary tangles, with enhancement of these processes following HSPG sulfation [4]. Within the CSF, elevation of several heparin-binding proteins such as SMOC1 and SPON1 have been shown to precede cognitive loss in AD by three decades [6–8]. A meta-analysis of several AD GWAS studies also revealed that heparan sulfate-glucosamine 3-sulfotransferase 1 gene (HS3ST1), the rate limiting step for heparan synthesis, is an AD risk locus [9].

Heparin, a hyper-sulfated form of heparan sulfate that is found in mast cells, is commonly used as an anti-coagulation treatment [10]. Unfractionated heparin (UFH) is a minimally processed form of naturally derived heparin and represented a major medical advance when introduced into clinical use in the 1930s. In the past three decades, low molecular weight heparin (LMWH) produced by an enzymatic depolymerization of UFH [11] have increasingly displaced UFH due to a favorable pharmacokinetics profile [10]. Both UFH and LMWH are frequently deployed for the treatment and prevention of thrombotic events such as atrial fibrillation (AF), deep vein thrombosis (DVT), and pulmonary embolism (PE) as well as to manage coagulation during procedures such as cardiac surgery, dialysis or extracorporeal circulation [10]. Specific binding targets vary with the GAG polysaccharide length but the important anticoagulant mechanism is achieved through binding of antithrombin (AT), inducing a conformational change that enhances AT activity by several orders of magnitude [12, 13], thus blocking the effects of endogenous coagulants thrombin and Factor Xa [14]. While the drug is commonly used as a short-term treatment and most forms are not thought to readily enter the brain, we postulated the exposure to heparin therapy might delay the clinical onset of AD.

Here we used Electronic Health Record (EHR) data from two different US health systems to conduct independent survival analyses of patients with and without a history of heparin therapy, at least 5 years of observation and subsequent diagnosis of possible AD dementia to test this hypothesis.

## Methods

### Study setting and data source

We extracted demographic data, diagnosis codes, and medication orders from both inpatient and outpatient visits of two health systems in New York City. Mount Sinai health system (MSHS) is a hospital network in New York City, USA. It was formed in September 2013 by merging the operations of Continuum Health Partners and the Mount Sinai Medical Center. We identified all consecutive admissions to five hospitals within the Mount Sinai health system. The Electronic Health Record (EHR) data were extracted from EPIC (Epic Systems Corporation, Verona WI). Final data retrieval was performed on July 12, 2022 and included all available records in the Mount Sinai EHR at that time.

Longitudinal EHR from New York Presbyterian (NYP) Columbia University Irving Medical Center (CUMC), located in the Washington Heights neighborhood of New York City, USA, were formatted using the OMOP common data model. Final data retrieval was performed on July 12, 2022 included all data in the NYP CUMC records since January 1st, 2000.

This retrospective cohort study received Institutional Review Board (IRB) approval from all participating institutions (MSHS: 19-00951, CUMC: AAAL0601). In both cases, the IRB committee waived informed consent.

### Study design

#### Population

Patients were included in the analyses if they had at least one inpatient encounter, were older than 65 by the end of the observation period and had at least five years of observation. For the MSHS site, we defined patients as having AD dementia if they had at least two recordings of “AD” following the International Classification of Diseases and Related Health Problems version 9 or 10 (ICD-9: 331.0, ICD-10: G30) or at least one record of an AD drug (Supplementary Table S1). For patients from the NYP CUMC site, AD dementia was defined as having two records of OMOP condition concept ID 378419 or a record of drug concept ID of 715997, 757627, or 733523.

#### Variables

We compared the age of AD dementia diagnosis between a heparin-exposed cohort (defined by OMOP drug concept id 21600972) and an unexposed cohort.

Covariates of interest included: age, sex, Charlson Comorbidity Index (CCI) [15] (following OHDSI guidelines [16]), number of heparin related comorbidities (defined as acute coronary syndrome, atrial fibrillation, deep venous thrombosis, and pulmonary embolism: Supplementary Table S2), number of inpatient admissions and follow up duration.

### Statistical analysis

Descriptive statistics were reported for all patient characteristics using means and standard deviation for age, medians with inter-quartile ranges for continuous variables, and counts with percentages for categorical variables. Continuous variables were compared using an unpaired t-test for two variables. Categorical variables were compared using the χ2 test.

The age of AD diagnosis was compared between patients with a record of heparin administration prior the AD diagnosis and patients without (t-test). Kaplan–Meier plots were created to present time to AD diagnosis in the different study populations.

All analyses were conducted with Python (Python software foundation, Version 3.6.5). Statistical significance was established at a 2-sided *P* < 0.05.

## Results

### Heparin administration is associated with later diagnosis among patients who progress to AD dementia in two independent cohorts

**Fig. 1:**
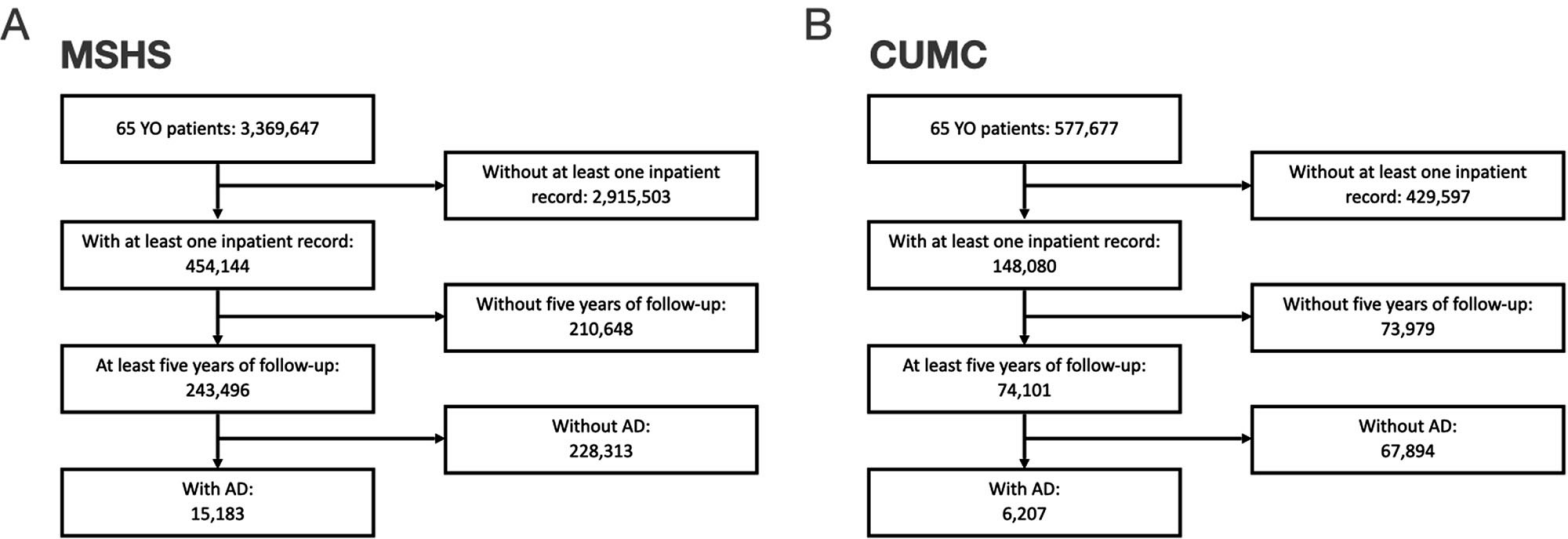
Subject selection criteria. Flow chart of subject selection for (**A**) MSHS, and (**B**) CUMC. MSHS Mount Sinai health system and CUMC Columbia University Irving Medical Center.

**Fig. 2:**
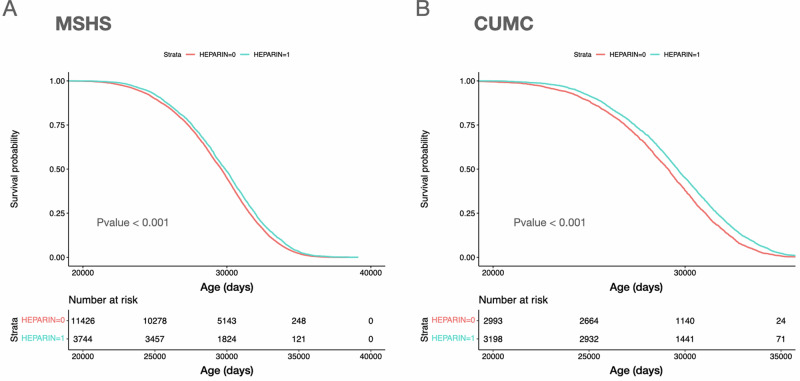
Association of heparin administration with AD diagnosis. Kaplan–Meier survival curves of age at diagnosis of AD among (**A**) MSHS, and (**B**) CUMC cohorts stratified by prior heparin administration. (Age, days) MSHS Mount Sinai health system and CUMC Columbia University Irving Medical Center.

To evaluate whether heparin therapy was associated with a potential protective effect upon eventual clinical diagnosis of AD dementia we performed a retrospective cohort study within the EHR of the Mount Sinai health system (MSHS). Our approach was to identify all individuals with a minimum age of 65 years at the time of the study, with at least five years of observation, and with a clinical diagnosis of AD (Total individuals *n* = 15,183, Fig. 1A, Table 1). We then compared patients with at least one heparin exposure prior to a diagnosis of AD dementia to patients unexposed to heparin (AD/Heparin(+): 24.7%, AD/Heparin(−): 75.3%). We observed that in general, AD/Heparin(+) subjects were of slightly younger mean age at the time of study than AD/Heparin(−) subjects (84.9 vs. 84.2 years), demonstrated a higher median Charlson Comorbidity Index (CCI) (4.0 vs. 3.0) and had an increased median duration of recorded clinical observation (10.0 vs. 9.0 years). We also observed that the age of clinical diagnosis of AD dementia was significantly later among AD/Heparin(+) (81.4 ± 8.6, IQR: 75.6–87.5) compared with AD/Heparin(−) (80.4 ± 8.6, IQR: 74.8–87.5) subjects (Fig. 2A, HR: 0.89, *P* value: 3.5e−10, univariate Cox regression, Supplementary Table 3). To evaluate whether heparin exposure remained significantly associated with a delayed AD dementia diagnosis while adjusting for potential confounding covariates we performed a multivariate Cox Proportional Hazards regression which also confirmed a protective hazard ratio when considering all AD subjects (HR: 0.60, *P* value: 5.2e−149, Supplementary Table 4) as well as in sex-stratified analyses, which revealed a protective hazard ratio in both females (HR: 0.57, *P* value: 1.0e−105, Supplementary Table 7) and males (HR: 0.64, *P* value: 1.8e−47, Supplementary Table 8).

**Table 1:** Mount Sinai Health System (MSHS) cohort characteristics.

	Entire cohort (*n* = 15,183)	Without prior heparin (*n* = 11438, 75.3%)	With prior heparin (*n* = 3745, 24.7%)	*P*-value
Age (years), median (IQR)	84.7 (78.5–90.3)	84.9 (78.7–90.4)	84.2 (77.9–89.9)	<0.001
Male, *N*. (%)	5779 (38.1)	4263 (37.3)	1516 (40.5)	<0.001
Charlson Comorbidity Index (CCI), median (IQR)	3.0 (2.0–6.0)	3.0 (2.0–5.0)	4.0 (2.0–7.0)	<0.001
Years of observation, median (IQR)	9.0 (7.0–13.0)	9.0 (7.0–13.0)	10.0 (7.0–15.0)	<0.001
Number of heparin-related comorbidities, median (IQR)	0.0 (0.0–1.0)	0.0 (0.0–1.0)	0.0 (0.0–1.0)	<0.001

We then sought to evaluate whether we also observed a later AD dementia diagnosis among AD/Heparin(+) subjects in an additional, independent cohort. We performed an equivalent retrospective cohort study within the EHR of the Columbia University Medical Center (CUMC). This cohort comprised 6,207 subjects with a clinical diagnosis of AD (AD/Heparin(+): 51.5%, AD/Heparin(−): 48.4%) selected according to identical criteria as described above (Fig. 1B, Table 2). We similarly observed that the age of clinical diagnosis of AD was significantly later among AD/Heparin(+) (80.7 ± 8.4, IQR: 74.9–86.7) compared with AD/Heparin(−) (78.9 ± 8.4, IQR: 73.5–85.1) subjects (Fig. 2B, HR: 0.80, *P* value: 3.55e−16, univariate Cox regression, Supplementary Table 5). Multivariate Cox Proportional Hazards regression revealed that heparin exposure was associated with a protective hazard ratio on the age of diagnosis of AD dementia when considering all AD subjects (HR: 0.69, *P* value: <2.2e−6, Supplementary Table 6) as well as in sex-stratified analyses, which revealed a protective hazard ratio in both females (HR: 0.63, *P* value: 9.5e−40, Supplementary Table 9) and males (HR: 0.76, *P* value: 6.3e−11, Supplementary Table 10).

**Table 2:** Columbia University Medical Center (CUMC) cohort characteristics.

	Entire cohort (*n* = 6207)	Without prior heparin (*n* = 3008, 48.5%)	With prior heparin (*n* = 3199, 51.5%)	*P*-value
Age (years), median (IQR)	84.4 (78.4–89.89)	84.56 (78.7–89.7)	84.3 (78.2–90.0)	0.811
Male, *N* (%)	2540 (40.9%)	1256 (41.8%)	1284 (40.1%)	0.204
Charlson Comorbidity Index (CCI), median (IQR)	1 (0–2)	0 (0–2)	1 (0–2)	0.004
Years of observation, median (IQR)	13.1 (8.83–17.8)	11.9 (8.15–16.6)	14.2 (9.72–18.6)	<0.001
Number of heparin-related comorbidities, median (IQR)	0 (0–1)	0 (0–1)	1 (0–1)	<0.001

## Discussion

In this short report we describe a retrospective cohort study performed on the EHRs of two large metropolitan hospital systems in the United States. Our study was aimed at determining whether clinical heparin usage received prior to the diagnosis of AD is associated with a difference in age of disease diagnosis and was motivated by recent compelling genetic and molecular findings that implicate HSPGs as potential mediators of the pathogenic and protective effects of APOE genotypes upon AD. In both cohorts we observed that heparin administration is associated with a significantly delayed diagnosis of AD.

Mechanistically, we postulate that heparin may function as a competitive inhibitor of the binding of APOE to endogenous HSPGs. We and our Colombian colleagues have recently shown that ApoE isoforms bind to heparin sulfate with differential affinity to HSPG (ApoE 4 > 3 > 2>>Christchurch) following our observation of resistance to AD dementia in an autosomal dominant AD mutation carrier with two copies of the rare APOE4 Christchurch mutation, suggesting a possible relationship between four levels of ApoE variant binding to heparan sulfate and the risk of AD [17]. We then generated an antibody to the APOE Christchurch binding domain and demonstrated its ability to inhibit ApoE binding to heparan sulfate, supporting the possibility of developing competitors of ApoE binding to HSPG that could be used in the treatment and prevention of AD.

Our study has several strengths. First, it illustrates the potential value of using large EHR data sets in a biologically informed way to help in the discovery of potentially repurposed drugs or, in this case, targets at which to aim in the discovery of drugs that might help in the treatment and prevention of AD, are safe and well tolerated, and reach their target in brain. We are not suggesting that heparin itself, a commonly short-term treatment with relatively limited blood brain barrier permeability and side effects, might be a preventive treatment for AD. Rather, we suggest that it may support the discovery of drugs that overcome these limitations and could eventually be put to the test in treatment and prevention trials. Second, findings were replicated in an additional independent EHR cohort.

Despite these potential strengths, the study has several limitations implicit in the use of EHR to identify interventions that associate with disease diagnosis, particularly the possibility that the intervention (heparin) is a proxy for improved healthcare (which could itself delay AD diagnosis) or is administered for conditions that may be protective factors for AD. It is worth noting that within both cohorts, AD/Heparin(+) subjects demonstrated a significantly increased burden of disease comorbidities (CCI) than AD/Heparin(−) subjects. CCI comprises a summary measure of overall disease burden formed from a weighted sum of major comorbid conditions and enables prediction of mortality rates [15]. Importantly, the CCI includes several conditions that are risk factors for AD, including type 2 diabetes mellitus [18], coronary heart disease [19], and cerebrovascular disease [20]. Similarly, several of the common indications for heparin include risk factors for AD, including atrial fibrillation [21], and coronary heart disease [19]. An additional likely confounder is the ApoE4 carrier status of the subjects within both EHR, which unfortunately is not known. In addition to being the main genetic driver of AD, ApoE4 is also a major risk factor for coronary atherosclerosis [22] and thus may represent an important source of potential confounding between AD and cardiovascular comorbidities that could necessitate heparin administration. Together, the associations between heparin associated comorbidities and AD/ApoE4 carriage indicate that although the set of AD/Heparin(+) and AD/Heparin(−) patients are likely confounded by differential disease burden, the biases might be expected to minimize the observation of any true protective effect of heparin upon AD diagnosis. This assertion is also consistent with the accentuation of the protective HR values we observed following the multivariate, compared with the univariate Cox regression analyses in both cohorts.

Our study is also limited in its adjustment of demographic covariates that could differ between AD/Heparin(+) and AD/Heparin(−) subjects. We note that the AD/Heparin(+) and AD/Heparin(−) groups demonstrate a significantly different composition of males and females in MSHS but not CUMC. The MSHS AD/Heparin(+) subjects are significantly younger (−0.7 years) than AD/Heparin(−) subjects, though this trend is not observed in the CUMC. We did not investigate differences in demographic factors such as education, socioeconomic status and race/ethnicity which could potentially drive a differential risk of AD diagnosis across heparin groups. In early iterations of our study, we observed a protective effect for heparin upon clinical AD diagnosis rates between age and comorbidity matched cohorts stratified for heparin exposure but were limited in our ability to adequately calibrate heparin exposure between AD cases (where we would ignore heparin exposures after clinical AD diagnosis) and subjects that were never observed to progress to AD and who might thus be artifactually enriched for heparin exposure. We also did not investigate relationships between frequency and dosage of heparin upon clinical AD diagnosis, nor evaluate whether the association is associated differentially with unfractionated or low molecular weight the heparin formulations, the latter being more likely to permeate the blood brain barrier [23].

An additional limitation of our study is the difficulty of accurately inferring phenotypes for a complex disorder such as AD, which requires the use of ICD and medication codes that are often optimized to assist medical billing rather than encoding complex medical phenomena. These limitations are partly obviated by the large numbers of patients contained within some EHR systems. Despite these limitations, the dual approach of using specific hypotheses informed by disease biology to guide analysis of real-world evidence that is capable of detecting even modest differences between clinical cohorts represents a powerful opportunity to reveal novel disease biology and potentially advance therapeutic discovery for this destructive disease.

In summary, we present findings of a potential protective effect of heparin administration upon eventual development of AD. While this study cannot delineate the causal structure of this association, these findings might inform the design of additional investigations to illuminate the association. Future studies that seek to verify these findings in additional cohorts (including APOE genotyped individuals), investigation of heparin dosage and duration responses, and profiling the effects of heparin administration in molecular studies of AD-model systems may offer avenues to advance these findings.

## Supplementary information

Supplementary Tables

## Data Availability

The data analyzed in this study are derived from electronic health records and are subject to privacy and legal restrictions. Due to the sensitive nature of the patient data and the confidentiality agreements in place, raw data cannot be made publicly available. However, aggregated data are summarized in the Supplementary Information available with this article.
