# Supplementary material for: Heparin treatment is associated with a delayed diagnosis of Alzheimer’s dementia in electronic health records from two large United States health systems
Source: Mol Psychiatry. 2024 Oct 8;30(4):1461–5. doi: 10.1038/s41380-024-02757-5 (PMC11919696; doi:10.1038/s41380-024-02757-5)
Supplement: Supplementary file 1 — Supplementary Tables [file 41380_2024_2757_MOESM1_ESM.docx]

**Supplementary Tables**

| **Table S1:** Medications associated with AD dementia classification |
| --- |
| **Medication** |
| Donepezil |
| Galantamine |
| Rivastigmine |
| Memantine |

| **Table S2:** Comorbidities associated with heparin administration | |
| --- | --- |
| **Diagnosis** | **OMOP concept IDs** |
| Acute Coronary Syndrome | 4215140 |
| Atrial Fibrillation | 313217 |
| Deep Venous Thrombosis (DVT) | 4133004 |
| Pulmonary Embolism (PE) | 440417 |

| **Table S3:**  Mount Sinai Health System (MSHS), Univariate Cox Proportional-Hazards Analysis | | | | | |
| --- | --- | --- | --- | --- | --- |
| **Covariate** | **Coef** | **Exp(Coef) / HR** | **Se(coef)** | **z** | **p** |
| **heparin_before** | -0.118336456 | 0.888397096 | 0.018863261 | -6.273382794 | 3.53287E-10 |

| **Table S4:**  Mount Sinai Health System (MSHS), Multivariate Cox Proportional-Hazards Analysis | | | | | |
| --- | --- | --- | --- | --- | --- |
| **Covariate** | **Coef** | **Exp(Coef) / HR** | **Se(coef)** | **z** | **p** |
| **heparin_before** | -0.51677 | 0.596442 | 0.019877 | -25.9981 | 5.2E-149 |
| **is_male** | -0.06927 | 0.933076 | 0.017056 | -4.06132 | 4.88E-05 |
| **age_at_start** | -0.42326 | 0.654906 | 0.002757 | -153.511 | 0 |
| **follow_up** | -0.40179 | 0.669122 | 0.003097 | -129.73 | 0 |
| **charlson_index** | 0.035968 | 1.036623 | 0.002799 | 12.84826 | 8.8E-38 |
| **comorb_count** | -0.05715 | 0.944457 | 0.015686 | -3.64315 | 0.000269 |
| **num_inpat** | 0.008824 | 1.008863 | 0.000684 | 12.89882 | 4.57E-38 |

| **Table S5:**  Columbia University Medical Center (CUMC), Univariate Cox Proportional-Hazards Analysis | | | | | |
| --- | --- | --- | --- | --- | --- |
| **Covariate** | **Coef** | **Exp(Coef) / HR** | **Se(coef)** | **z** | **p** |
| **heparin_before** | -0.2253 | 0.7983 | 0.0256 | -8.799 | <2e-16 |

| **Table S6:**  Columbia University Medical Center (CUMC), Multivariate Cox Proportional-Hazards Analysis | | | | | |
| --- | --- | --- | --- | --- | --- |
| **Covariate** | **Coef** | **Exp(Coef) / HR** | **Se(coef)** | **z** | **p** |
| **heparin_before** | -0.377368 | 0.685663 | 0.026741 | -14.112 | < 2e-16 |
| **is_male** | -0.087788 | 0.915955 | 0.026485 | -3.315 | 0.000918 |
| **age_at_start** | -0.324231 | 0.723083 | 0.003463 | -93.63 | < 2e-16 |
| **follow_up** | -0.283004 | 0.753517 | 0.003986 | -70.991 | < 2e-16 |
| **charlson_index** | 0.053743 | 1.055213 | 0.008163 | 6.584 | 4.58E-11 |
| **comorb_count** | 0.127811 | 1.136339 | 0.022913 | 5.578 | 2.43E-08 |
| **num_inpat** | -0.037843 | 0.962864 | 0.019951 | -1.897 | 0.057853 |

| **Table S7:**  Mount Sinai Health System (MSHS), Multivariate Cox Proportional-Hazards Analysis, Female Only | | | | | |
| --- | --- | --- | --- | --- | --- |
| **Covariate** | **Coef** | **Exp(Coef)**  / **HR** | **Se(coef)** | **z** | **p** |
| **heparin_before** | -0.56731 | 0.567051 | 0.025978 | -21.8378 | 1E-105 |
| **age_at_start** | -0.43238 | 0.648961 | 0.003802 | -113.736 | 0 |
| **follow_up** | -0.41038 | 0.663398 | 0.004151 | -98.8687 | 0 |
| **charlson_index** | 0.040058 | 1.040871 | 0.003682 | 10.88059 | 1.43E-27 |
| **comorb_count** | -0.0408 | 0.960025 | 0.020626 | -1.97794 | 0.047935 |
| **num_inpat** | 0.00876 | 1.008798 | 0.000898 | 9.753437 | 1.78E-22 |

| **Table S8:**  Mount Sinai Health System (MSHS), Multivariate Cox Proportional-Hazards Analysis, Male Only | | | | | |
| --- | --- | --- | --- | --- | --- |
| **Covariate** | **Coef** | **Exp(Coef)**  / **HR** | **Se(coef)** | **z** | **p** |
| **heparin_before** | -0.44929 | 0.638082 | 0.031042 | -14.4737 | 1.78E-47 |
| **age_at_start** | -0.41356 | 0.661292 | 0.004122 | -100.341 | 0 |
| **follow_up** | -0.39268 | 0.675244 | 0.004855 | -80.887 | 0 |
| **charlson_index** | 0.029709 | 1.030155 | 0.004333 | 6.857175 | 7.02E-12 |
| **comorb_count** | -0.06878 | 0.933529 | 0.02427 | -2.83407 | 0.004596 |
| **num_inpat** | 0.008915 | 1.008955 | 0.001061 | 8.404903 | 4.28E-17 |

| **Table S9:**  Columbia University Medical Center (CUMC), Multivariate Cox Proportional-Hazards Analysis, Female Only | | | | | |
| --- | --- | --- | --- | --- | --- |
| **Covariate** | **Coef** | **Exp(Coef)**  / **HR** | **Se(coef)** | **z** | **p** |
| **heparin_before** | -0.46370072 | 0.6289518 | 0.035144399 | -13.1941568 | 9.48E-40 |
| **age_at_start** | -0.3088622 | 0.7342819 | 0.004314362 | -71.5893014 | 0.00E+00 |
| **follow_up** | -0.26309746 | 0.768667 | 0.005053195 | -52.0655625 | 0.00E+00 |
| **charlson_index** | 0.05935624 | 1.0611532 | 0.011098506 | 5.3481286 | 8.89E-08 |
| **comorb_count** | 0.19613295 | 1.2166886 | 0.02968347 | 6.6074804 | 3.91E-11 |
| **num_inpat** | 0.00384212 | 1.0038495 | 0.025817925 | 0.148816 | 8.82E-01 |

| **Table S10:**  Columbia University Medical Center (CUMC), Multivariate Cox Proportional-Hazards Analysis, Male Only | | | | | |
| --- | --- | --- | --- | --- | --- |
| **Covariate** | **Coef** | **Exp(Coef)**  / **HR** | **Se(coef)** | **z** | **p** |
| **heparin_before** | -0.27025469 | 0.7631851 | 0.041342255 | -6.5370089 | 6.28E-11 |
| **age_at_start** | -0.36300292 | 0.6955844 | 0.006310874 | -57.5202297 | 0.00E+00 |
| **follow_up** | -0.32803905 | 0.7203349 | 0.007029653 | -46.6650387 | 0.00E+00 |
| **charlson_index** | 0.0552713 | 1.0568273 | 0.012230218 | 4.5192406 | 6.21E-06 |
| **comorb_count** | 0.02102848 | 1.0212511 | 0.035969586 | 0.5846183 | 5.59E-01 |
| **num_inpat** | -0.13018277 | 0.877935 | 0.031608776 | -4.118564 | 3.81E-05 |

| **Table S11:** Mount Sinai Health System (MSHS), Heparin Formulations Administered | |
| --- | --- |
| **Medication** | **# Patients** |
| HEPARIN | 6678 |
| ENOXAPARIN | 1522 |
| LOVENOX | 30 |
| FONDAPARINUX | 7 |
| DALTEPARIN | 1 |

| **Table S12:** Columbia University Medical Center (CUMC), Heparin Formulations Administered | |
| --- | --- |
| **Medication** | **# Patients** |
| HEPARIN | 2288 |
| ENOXAPARIN | 53 |
| LOVENOX | 981 |
| FONDAPARINUX | 0 |
| DALTEPARIN | 49 |
